# Inefficiency of Kocher and Caird’s Criteria in Septic Arthritis of the Hip Due to *Kingella kingae*: A Multicenter Retrospective Cohort Study

**DOI:** 10.3390/microorganisms13102323

**Published:** 2025-10-07

**Authors:** Giacomo De Marco, Oscar Vazquez, Blaise Cochard, Piotr Foland, Ulrich Meinzer, Cindy Mallet, Brice Ilharreborde, Edouard Haumont, Zagorka Pejin-Arroyo, Pablo Yagupsky, Amadeu Gené, Eneritz Velasco Arnaiz, Catarina Gouveia, Joana Arcangelo, Nicolas Mainard, Jocelyn Gravel, Tony Walls, Nienke Hagedoorn, Ameneh Khatami, Mohamed Tashani, Silvia Valisena, Christina Steiger, Romain Dayer, Moez Chargui, Rosa Maria Alcobendas Rueda, Elisa Fernandez-Cooke, Beatriz Bravo, Laura Martin Pedraz, Sara Murias Loza, Daniel Clemente, Federico Canavese, Dimitri Ceroni

**Affiliations:** 1 University Hospitals of Geneva, 1205 Geneva, Switzerlandoscar.vazquez@hug.ch (O.V.);; 2 Department of General Pediatrics, Pediatric Internal Medicine, Rheumatology and Infectious Diseases, National Reference Centre for Rare Pediatric Inflammatory Rheumatisms and Systemic Autoimmune Diseases (RAISE), CHU Robert Debré, AP-HP, Université Paris Cité, 75019 Paris, France; 3 Pediatric Orthopedic Department, CHU Robert Debré, AP-HP, Université Paris Cité, 75019 Paris, France; 4 Service de Chirurgie Orthopédique Pédiatrique, Hôpital Necker-Enfants-Malades, 75015 Paris, France; 5 Soroka University Medical Center, Beer-Sheva 84101, Israel; 6 Hospital Sant Joan de Déu, 08950 Barcelona, Spain; 7 Pediatric Infectious Diseases Unit, Hospital Dona Estefânia, ULS São José, Academic Clinical Centre of Lisbon, 1169-050 Lisbon, Portugal; 8 Comprehensive Health Research Centre (CHRC), Nova Medical School, Faculdade Ciências Médicas, 1169-050 Lisbon, Portugal; 9 Pediatric Orthopedics Unit, Hospital Dona Estefânia, ULS São José, Academic Clinical Centre of Lisbon, 1169-050 Lisbon, Portugal; 10 Department of Pediatric Orthopedic Surgery, Faculty of Medicine, Lille University Hospital, 59037 Lille, France; 11 Department of Pediatrics, Sainte-Justine Hospital, University of Montreal, Montreal, QC H3T 1C5, Canada; 12 Department of Pediatrics and Child Health, University of Otago, Christchurch 8140, New Zealand; 13 Department of General Pediatrics, Erasmus MC Sophia Children’s Hospital, 3015 GD Rotterdam, The Netherlands; 14 Department of Infectious Diseases and Microbiology, The Children’s Hospital at Westmead, Sydney, NSW 2145, Australia; 15 Sydney Infectious Diseases Institute, University of Sydney, NSW 2145, Australia; 16 Specialty of Child and Adolescent Health, The Children’s Hospital at Westmead, University of Sydney, Sydney, NSW 2145, Australia; 17 Hôpital de Neuchâtel, 2000 Neuchâtel, Switzerland; 18 Hospital Universitario La Paz, 28041 Madrid, Spain; 19 Hospital Universitario 12 de Octubre, imas12, 28041 Madrid, Spain; 20 Hospital Universitario Virgen de las Nieve, 18014 Granada, Spain; 21 Materno-Infantil, 29011 Malaga, Spain; 22 Hospital Universitario Central de Asturias, 33011 Oviedo, Spain; 23 Hospital Infantil Universitario Niño Jesús, 28009 Madrid, Spain

**Keywords:** septic arthritis, *Kingella kingae*, hip joint, children, *Kocher & Caird* criteria, algorithm, pediatric infections

## Abstract

In children under 4, septic arthritis of the hip (SAH) caused by *Kingella kingae* (SAH-KK) can be misdiagnosed, as it does not meet classic septic joint criteria (fever > 38.5°, pain, limited range of motion, and inability to bear weight). The objective of this study was to report clinical and paraclinical characteristics in a large cohort of children with confirmed SAH-KK and to evaluate the reliability of the Kocher (KC) and Caird criteria (CC) in predicting SAH-KK. Medical records of 140 children with confirmed SAH-KK were collected. Data on sex, age, temperature on admission, weight-bearing status, white blood cell (WBC) count, platelet count, C-reactive protein (CRP) value, and erythrocyte sedimentation rate (ESR) were extracted. The study focused on the sensitivity of KC (body temperature, refusal to bear weight, leukocytosis, and ESR) and CC (KC criteria plus CRP level). All patients had bacteriologically confirmed SAH-KK; most had mild symptoms and near-normal inflammatory markers. CRP (76.2%) had the highest sensitivity, followed by weight-bearing status (73.8%) and WBC count (69.6%). Body temperature and ESR exceeded cutoff values in less than 50% of cases. Among 77 patients fulfilling all KC, 49 (63.5%) had less than a 40% probability of SAH. Of 50 children with complete CC, 20 (40%) had a 62.4% or lower probability of SAH. KC and CC are not sufficiently accurate to confidently exclude SAH-KK in preschool-aged children due to heterogeneous clinical presentations. Further studies are needed to redefine diagnostic criteria based on patient age and causative pathogens.

## 1. Introduction

Limping in the pediatric population is a common reason for seeking medical attention. In this regard, the exact incidence of atraumatic limping in children remains difficult to estimate, but the few data available in the literature indicate an incidence ranging from 1.8 to 2.8 children out of 1000 [1,2]. Another report even affirms that 5% of emergency department visits are motivated by limp as the chief complaint [3]. Several conditions can be responsible for gait disturbance, presenting a real diagnostic challenge. Transient synovitis of the hip (TSH) is a benign and self-limited condition that primarily affects children between the ages of 4 and 10 years and is one of the most frequent causes of acute limping in children [4]. It is, therefore, tempting to assign this diagnosis to all patients presenting with a limp who do not have evident clinical or biological findings classically described in joint infections. On the other hand, septic arthritis of the hip (SAH) is a leading cause of hip pain and limping in this age group. If misdiagnosed, SAH can lead to serious complications, including joint destruction, femoral head necrosis, and permanent joint disability [5,6,7,8,9,10,11,12,13,14,15].

To improve the early detection of SAH, an algorithm combining clinical and laboratory findings [body temperature > 38.5 °C, inability to bear weight, white blood cell (WBC) count > 12,000 cells/mm^3^, and erythrocyte sedimentation rate (ESR) > 40 mm/h] was developed by Kocher et al. in 1999 [16] and validated by the same authors in 2003 [17]. The algorithm was later modified by Caird et al. [18] with the addition of C-reactive protein (CRP) levels > 20 mg/L [16,17,18]. The likelihood of SAH is directly related to the number of positive predictors, with TSH classically characterized by low scores [16,17,18]. The accuracy of these algorithms has been evaluated and confirmed in many studies involving pediatric patients older than 4 years with bacterial arthritis caused by pyogenic traditional pathogens, such as *Staphylococcus aureus*, *Streptococcus pyogenes*, and *Streptococcus pneumoniae* [16,17,18,19].

*K. kingae* is a facultative anaerobic, β-hemolytic, Gram-negative organism that is notoriously difficult to grow on routine solid cultures of blood or body fluids, such as bone exudates or synovial fluid [20]. *K. kingae* is currently considered the main bacterial cause of osteoarticular infections (OAIs), especially in children under 48 months of age [21,22]. Regardless of the infection type, *K. kingae* OAIs are typically characterized by mild clinical presentations and modest inflammatory responses, often resulting in few symptoms evocative of bacterial infections [21,22].

Since the early 2000s, the advent of nucleic acid amplification assays (NAAAs) has significantly reduced the proportion of culture-negative osteoarticular infections, providing irrefutable evidence that *K. kingae* was the most common pathogen responsible for osteoarticular infection in children under the age of 4 [21,22,23,24]. However, clinical and laboratory features of septic arthritis due to *K. kingae* are usually unremarkable, requiring a high index of suspicion [25,26,27]. In this regard, Yagupsky et al. alerted the scientific community that the *Kocher* predictive algorithm was not sensitive enough to detect SAH caused by *K. kingae* (SAH-KK) [28]. Hagedoorn et al. confirmed that both the *Kocher* and *Caird* predictive algorithms lack sensitivity to rule out septic hip arthritis in the early assessment of preschool-aged children with acute hip pain [29]. Valisena et al. suggested in a recent narrative review that the Kocher and Caird criteria were still at the center of a debate on the diagnostic tools used for SAH in children less than 4 years, and they openly questioned the validity of predictive algorithms, especially when *K. kingae* was the causative pathogen [30].

The purpose of this study is to report the clinical and paraclinical characteristics of a large cohort of children under 4 years old with confirmed SAH caused by *K. kingae* (SAH-KK). The second objective is to evaluate the reliability of the *Kocher* criteria (KC) and the *Caird* criteria (CC) in predicting SAH-KK by estimating the sensitivity of the different items in the two algorithms.

## 2. Materials and Methods

A multicenter retrospective cohort study reviewed the medical charts of children treated for SAH (with or without associated osteomyelitis) in 17 hospitals from 7 countries (Switzerland (2), France (3), Spain (7), Portugal (1), Israel (1), Canada (1), New Zealand (1), and Australia (1)). We focused on children with confirmed SAH-KK with positive blood or joint fluid cultures and/or positive blood/joint fluid NAAAs for *K. kingae*.

Sex, age, temperature on admission, weight-bearing status, WBC count, CRP value, and ESR were extracted from the medical charts for each patient. The KC and CC were used as cutoffs to assess the likelihood of SAH [16,18]. When available, the platelet count was also collected since it has been demonstrated that this parameter could be disrupted in children with invasive infection due to *K. kingae* [26,31].

Blood cultures and joint aspirate samples were sent to the laboratory for immediate inoculation before starting antibiotic therapy. Two polymerase chain reaction (PCR) assays, namely, broad-range PCR and *K. kingae*-specific real-time PCR, were performed on joint aspiration samples for bacterial identification when standard cultures were negative.

Patients were excluded if they had no bacteriological confirmation of *K. kingae*, had no available laboratory data, or had not been treated with antibiotics. Principal ethical approval was obtained from the Cantonal Research Ethics Commission of the Canton of Geneva (CER 2023-00578), the lead center, as well as for the other centers.

Demographics and clinical characteristics were expressed as medians, ranges, means, and standard deviations for continuous outcomes and frequencies with percentages for categorical outcomes. Children with complete data were used to assess the accuracy of the Kocher and Caird algorithms for predicting SAH-KK. Demographic and clinical comparisons between cases with complete vs. incomplete data were performed using a paired Student’s *t*-test. For each variable in the *Kocher* and modified *Caird* criteria, we reported the number of patients meeting the criteria and the sensitivity (calculated based on patients with complete data) for detecting confirmed *K. kingae* septic arthritis of the hip. Statistical analyses were performed using the Jamovi software, version 2.3 (The Jamovi Project (2022), accessed on 25 May 2023 at https://www.jamovi.org).

## 3. Results

### 3.1. Patient Characteristics and Demographics (Table 1)

One hundred and forty children with a confirmed diagnosis of SAH-KK were enrolled in the study. Associated osteomyelitis was observed in two children (1.4%); all remaining patients presented with isolated SAH. The mean age at diagnosis was 16.8 +/− 6.4 months (median value 14 months), with a significant male predominance (61.6%; *p* < 0.01); children between 0 and 12 months and those between 13 and 24 months were overrepresented in the study’s cohort, reaching 50 (35.7%) and 70 (50%) cases, respectively. Overall, 116 (82.9%) children were younger than 2 years, and only 2 were older than 4 years (Table 1).

**Figure 1:**
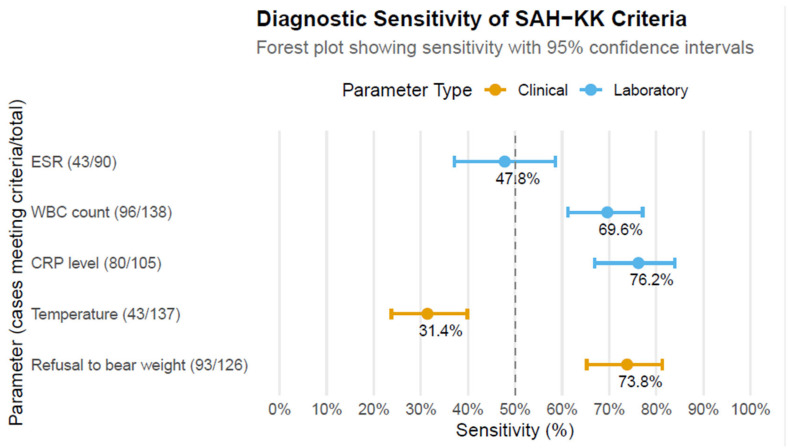
Sensitivity of clinical and laboratory variables of the *Kocher* and modified *Caird* criteria for detecting patients with confirmed septic arthritis of the hip due to *K. kingae*. Note: Sensitivity estimates are presented descriptively without statistical comparison between parameters due to differing sample sizes and the study’s observational design.

The mean and median temperatures on admission were 38.1 °C and 38.2 °C, respectively; only 43 of 137 patients (31.4%) presented with a body temperature >38.5 °C (Figure 1). Temperature data were missing in three cases; in six cases, the only information available was that the temperature was higher or lower than 38.5 °C. Weight-bearing status was available for 126 patients. In the remaining 14 patients, this information was not documented, or the patient was still unable to walk due to their young age. WBC count analysis was available in 138 patients with a mean value of 14,614/mm^3^ +/− 3643/mm^3^. In 69.6% of the cases, the WBC count was higher than 12,000/mm^3^, while in 26.1% of the patients, the WBC count was higher than 17,000/mm^3^. Data on CRP and ESR results were incomplete due to the multicentric origin of the information and the different diagnostic practices of the participating centers. ESR information was available for 90 patients (64.3%), CRP was available for 105 patients (75%), and both CRP and ESR were available only for 65 patients (46.4%). The median CRP level reached 35 mg/L, while CRP >20 mg/L was observed in 76.2% of patients (80/105). The median ESR was 37.5 mm/h, and 43 of 90 (47.8%) of patients had an ESR >40 mm/h. Finally, platelet counts were higher than 400,000/m^3^ in 49 of 75 (65.3%) patients (mean value 441,030/mm^3^ +/− 57,276/m^3^).

### 3.2. Kocher Criteria and Caird Criteria (Table 2)

In this cohort of patients with SAH-KK, 77 of the 140 patients (56.7%) had the four KC, whereas only 50 (30%) of them had all five CC (Table 2). Sensitivity analysis comparing demographic and clinical characteristics between cases with complete vs. incomplete data (*p* > 0.05) confirmed that missing data were random rather than systematic. Globally, the median Kocher score was 2 (IQR 1–3), while the median Caird score was 3 (IQR 1–4). According to the predictive values of the Kocher score, 63.6% of patients with proven SAH-KK had less than a 40% probability of suffering SAH. Furthermore, according to the Caird score, 40% of children in our cohort with proven *K. kingae* infections had a 62.4% or smaller probability of experiencing SAH.

## 4. Discussion

The results of the present study highlight that neither the KC nor the CC are sufficiently accurate to confidently exclude SAH-KK in preschool-aged children. *K. kingae* osteoarticular infections (OAIs) are often characterized by a mild clinical presentation and limited inflammatory response, resulting in patients with few or no predictive diagnostic criteria [21,22,23,26,27] and poorly predictive decision-making algorithms [28,29]. The present multicentric study includes the largest series of SAH-KK to evaluate the utility of KC and CC for the early detection of SAH patients younger than 4 years of age.

Previous work has shown that most SAH-KK infections occur in children younger than 4 years of age. Our cohort of patients, although retrospective in nature, is no exception, with only 2/140 children older than 4 years at the time of diagnosis. To explain this, it is important to remember that *K. kingae* oropharyngeal colonization is most prevalent in children up to the age of 4 years [32]. It is recognized in this regard that *K. kingae* oropharyngeal colonization and carriage play a crucial role in the person-to-person transmission of the bacterium and its dissemination in the community, actively contributing to the pathogenesis of invasive infections.

Furthermore, it has been shown that maternal immunity transmitted to the fetus during pregnancy gradually declines in early childhood, especially between 6 and 24 months [27,33].

The present results also confirmed that children with SAH-KK present inconsistently with a high fever. Only 42% of the patients in this series had a temperature higher than 38.5° on admission, and the absence of temperature cannot be considered a factor excluding infection.

In the current series, CRP (76.2%) had the greatest sensitivity in detecting SAH-KK, followed by weight-bearing refusal (73.8%) and WBC count (69.6%), whereas body temperature and ESR levels exceeded the cutoff values of the Kocher and Caird algorithms in less than 50% of cases. Therefore, it appears that CRP is the better biological marker for assessing SAH-KK. CRP was added to the Kocher algorithm only in 2006 by Caird et al., and a threshold of 20 mg/L was adopted [18,34,35]. The median CRP level in our cohort was 35 mg/L, and CRP levels were higher than 20 mg/L in more than 75% of children with SAH-KK. These values were consistent with those published in previous studies; in fact, a previous study by Ilharreborde et al. described a mean CRP level of 39 mg/L in a cohort of 31 children with *K. kingae* septic arthritis [24]. Dubnov-Raz et al. also demonstrated that CRP levels reached a mean of 37 mg/L in a large series of septic arthritis caused by *K. kingae* [33]. However, other conditions, including viral infections and inflammatory conditions that may lead to mild elevations in CRP, usually reach up to 25 mg/L and 50 mg/L, respectively [26]. In this regard, since there is uncertainty regarding the definition of its cutoff value, it may be reasonable to reconsider the threshold for CRP in SAH-KK with information that is not based on a prospective comparative study.

Our results showed that ESR values were also of limited value for the diagnosis of SAH-KK and needed to be interpreted with caution. In fact, the median ESR in our study was 37.5 mm/h, in agreement with previous studies focusing on *K. kingae* septic arthritis. In fact, similar results were found in a study by Dubnov-Raz et al. about *K. kingae* OAIs, in which ESR reached 44.1 mg/L in cases of septic arthritis [27,33]. Only 47.8% of our patients with confirmed SAH-KK had an ESR rate higher than 40 mm/h. ESR is, therefore, a poor predictor of SAH-KK, although this parameter was previously considered to be one of the most sensitive and most easily obtained paraclinical tests for detecting infection [29].

Our results suggest that the platelet count could become a useful additional parameter for detecting SAH-KK since 65.3% of the patients of this series (n = 49/75) had a platelet count higher than 400,000/mm^3^. Platelets contribute to the systemic inflammatory and immune response and enhance the host’s defense against pathogens [36]. Acute bacterial infection usually inhibits platelet production, whereas chronic inflammation is often associated with reactive thrombocytosis [36,37,38]. In a previous study, Coulin et al. showed a statistical difference (*p* < 0.001) in the platelet count between patients with S. aureus and *K. kingae* OAI [31]. Nowadays, it is widely accepted that most *K. kingae* OAIs may present with an elevated platelet count, even in the absence of fever or an elevated WBC count [26,31]. This reactive thrombocytosis is probably due to *K. kingae* infections taking longer to manifest clinically than classic pyogenic infections owing to the low virulence of this pathogen [36,37,38].

More interestingly, this study confirmed that the *Kocher* and *Caird* predictive algorithms are not sufficiently accurate to exclude the diagnosis of SAH-KK in preschool children. Application of the KC algorithm to our results suggested that the probability of bacterial arthritis was less than 40% (two or fewer criteria) in 49 (63.6%) of the patients fulfilling all criteria. The probability of SAH was estimated to be equal to or less than 62.4% in 40% of the 50 children with completely available CC.

We have encountered several limitations in the analysis of our results. The first limitation of our study is represented by the selection bias of the KC and CC predictors themselves in defining SAH-KK. These scores depend on both specific conditions, which are extremely dependent on the patient’s age.

A second limitation lies in the study’s design. Missing values for key variables (CRP, ESR, and platelet counts) reduce statistical power. The multicenter retrospective design of this study inevitably led to missing values for key variables (CRP, ESR, and platelet counts). Thus, this can interfere with the data interpretation and reduce statistical power. However, the consistent pattern across all evaluated parameters (with CRP, weight-bearing status, and WBC count showing the highest sensitivity despite missing values) strengthens our conclusion that current criteria are inadequate for SAH-KK diagnosis. In addition, the diagnosis of SAH-KK was made by different decision algorithms, according to the clinical practice of each participating hospital. For this reason, many incomplete cases had to be excluded from the assessment of the KC and CC algorithms, potentially affecting the robustness of our analysis. However, sensitivity analyses comparing demographic and clinical characteristics between cases with complete versus incomplete data showed no significant differences (*p* > 0.05), suggesting that missing data were random rather than systematic in nature. This finding supports that our excluded cases were unlikely to bias the observed diagnostic performance of the algorithms.

Third, both the Kocher and Caird algorithms do not take into account that the cutoff for abnormal WBC values in infants and children under 4 years of age (>17,500/mm^3^ in children 6–12 months of age; >17,000/mm^3^ in children 13–24 months of age >14,500/mm^3^ in children 24–48 months of age [26]) is significantly higher than 12,000/mm^3^. Using age-related WBC count cutoffs, less than 30% of patients with SAH-KK would have abnormal results for the WBC count predictor in the algorithm. All of this indicates that the WBC count threshold in both algorithms appears to be inappropriate when applied to the age group in which OAIs due to *K. kingae* occur.

Finally, the absence of a control group of septic arthritis of the hip with other pathogens and a control group with medical conditions that could cause limping made it impossible to estimate the specificity of the different criteria included in the algorithms. On the other hand, the sensitivity of the different items in the two algorithms could be assessed since only true positives were involved.

## 5. Conclusions

Neither the KC nor the CC are sufficiently accurate to confidently exclude SAH-KK in preschool-aged children because of its heterogeneous clinical presentation. Our results also highlight the need for new and improved algorithms based on clinical and biological predictors that could be applied to SAH-KK in children younger than 4 years.

Currently, we are primarily using the oropharyngeal *K. kingae* PCR, a diagnostic test recommended in the PIDS guidelines, to increase diagnostic sensitivity. In addition, two fairly specific markers of these infections are currently used, namely, ESR and platelet count.

Lastly, we expect that the solution will be brought to clinicians with the advent of high-throughput sequencing, namely, metagenomic Next-Generation Sequencing (mNGS). This new technology is beginning to be used in clinical practice, but beyond its classic use on biopsy samples, it is legitimate to wonder whether future diagnoses will not be made indirectly on plasma samples since the method is able to detect not only pathogens circulating in the bloodstream but also those emanating from focal infections.

Further studies seem necessary to redefine the diagnostic criteria according to the age of the patients and the causative pathogens that can clearly distinguish SAH-KK and SAH from other pathogens and benign conditions, such as TSH.

## Figures and Tables

**Table 1:** Demographics of the patients with confirmed septic arthritis of the hip due to *K. kingae*.

Variables	Values *(n* = 140)
Gender, *n* (%)	
Female	39 (36.8%)
Male	67 (63.2%)
Not reported	34
Age, months, mean (SD) [median, range]	16.8 (6.4) [14; 4–56]
Age repartition, *n* (%)	
0–12 months	50 (35.7%)
13–24 months	70 (50.0%)
25–36 months	11 (7.9%)
37–48 months	7 (5.0%)
>48 months	2 (1.4%)

**Table 2:** Summary of the *Kocher* and the *Caird* algorithms in patients with confirmed *K. kingae*’s septic arthritis of the hip.

Variables	Kocher Algorithm (*n* = 4 Criteria *)	Caird Algorithm (*n* = 5; KC + CRP Level **)
Patients with complete data	77/140 (56.7%)	50/140 (30.0%)
Mean (SD) score	2 (1.1)	2.8 (1.3)
Median score	2	3
Range	0–4	0–5
Number of present criteria		
0	4 (5.2%)	2 (4%)
1	18 (23.4%)	9 (18%)
2	27 (35%)	9 (18%)
3	23 (29.9%)	10 (20%)
4	5 (6.5%)	17 (34%)
5	-	3 (6%)

* Kocher criteria (KC): body temperature of >38.5 °C; inability to bear weight; WBC > 12,000 leukocytes/mm^3^; ESR > 40 mm/h. ** Caird criteria: KC plus CRP > 20 mg/L.

## Data Availability

The data presented in this study are available upon request from the corresponding author. The data are not publicly available due to privacy concerns.

## Abbreviations

The following abbreviations are used in this manuscript:

*K. kingae*	*Kingella kingae*
SA	Septic arthritis
SAH	Septic arthritis of the hip
SAH-KK	Septic arthritis of the hip caused by *K. kingae*
TSH	Transient synovitis of the hip
OAI	Osteoarticular infection
WBC	White blood cell
ESR	Erythrocyte sedimentation rate
CRP	C-reactive protein
